# Murine Models for the Study of Fetal Alcohol Spectrum Disorders: An Overview

**DOI:** 10.3389/fped.2020.00359

**Published:** 2020-07-15

**Authors:** Laura Almeida, Vicente Andreu-Fernández, Elisabet Navarro-Tapia, Rosa Aras-López, Mariona Serra-Delgado, Leopoldo Martínez, Oscar García-Algar, María Dolores Gómez-Roig

**Affiliations:** ^1^Maternal and Child Health and Development Network II (SAMID II), Instituto de Salud Carlos III (ISCIII), Barcelona, Spain; ^2^Fundació Sant Joan de Déu, Barcelona, Spain; ^3^BCNatal Barcelona Center for Maternal Fetal and Neonatal Medicine, Hospital Sant Joan de Déu and Hospital Clínic, Barcelona, Spain; ^4^Nutrition and Health Deparment, Valencian International University (VIU), Valencia, Spain; ^5^Grup de Recerca Infancia i Entorn (GRIE), Institut D'investigacions Biomèdiques August Pi i Sunyer (IDIBAPS), Barcelona, Spain; ^6^Congenital Malformations Lab, Institute of Medicine and Molecular Genetic (INGEMM), Institute for Health Research of La Paz Universitary Hospital (IdiPAZ), Madrid, Spain; ^7^Department of Pediatric Surgery, Hospital Universitario La Paz, Madrid, Spain; ^8^Department of Neonatology, Hospital Clínic-Maternitat, ICGON, IDIBAPS, BCNatal, Barcelona, Spain

**Keywords:** prenatal alcohol exposure, fetal alcohol spectrum disorders, fetal alcohol syndrome, alcohol consumption patterns, facial dysmorphology, neurodevelopmental disorders, fetal growth restriction, models of fetal alcohol spectrum disorders

## Abstract

Prenatal alcohol exposure is associated to different physical, behavioral, cognitive, and neurological impairments collectively known as fetal alcohol spectrum disorder. The underlying mechanisms of ethanol toxicity are not completely understood. Experimental studies during human pregnancy to identify new diagnostic biomarkers are difficult to carry out beyond genetic or epigenetic analyses in biological matrices. Therefore, animal models are a useful tool to study the teratogenic effects of alcohol on the central nervous system and analyze the benefits of promising therapies. Animal models of alcohol spectrum disorder allow the analysis of key variables such as amount, timing and frequency of ethanol consumption to describe the harmful effects of prenatal alcohol exposure. In this review, we aim to synthetize neurodevelopmental disabilities in rodent fetal alcohol spectrum disorder phenotypes, considering facial dysmorphology and fetal growth restriction. We examine the different neurodevelopmental stages based on the most consistently implicated epigenetic mechanisms, cell types and molecular pathways, and assess the advantages and disadvantages of murine models in the study of fetal alcohol spectrum disorder, the different routes of alcohol administration, and alcohol consumption patterns applied to rodents. Finally, we analyze a wide range of phenotypic features to identify fetal alcohol spectrum disorder phenotypes in murine models, exploring facial dysmorphology, neurodevelopmental deficits, and growth restriction, as well as the methodologies used to evaluate behavioral and anatomical alterations produced by prenatal alcohol exposure in rodents.

## Introduction

Alcohol is a known teratogen. Its frequent use during pregnancy impacts the normal development of human fetuses promoting severe developmental alterations and generating a wide range of physical, behavioral, cognitive, and neurological impairments. In 1968, Lemoine et al. established an association between prenatal alcohol exposure (PAE) with certain neurodevelopmental disabilities (1). However, it was not until 1973 when Jones and Smith provided the initial characterization of fetal alcohol syndrome (FAS) (2), defined as growth restriction, facial dysmorphologies (wide-spaced eyes, mid-facial hypoplasia, and a smooth philtrum), and central nervous system (CNS) disorders, resulting in motor, cognitive and behavioral disorders (3). Subsequent observational studies identified and characterized the umbrella term fetal alcohol spectrum disorder (FASD) (4) that includes: FAS (the most deleterious manifestation of FASD), partial FAS (pFAS) (an intermediate phenotype defined by the absence of some FAS characteristics), alcohol-related birth defects (ARBD) (certain physical impairments are exhibited), and alcohol-related neurological disorders (ARND) (behavioral and learning neuropsychological alterations, usually without facial dysmorphology) (5).

Thus, behavioral deficits in FASD subjects associate with structural changes in brain organogenesis: the *Corpus callosum* may lose its structure (agenesis) and generate cognitive deficits linked to attention, executive and psychosocial functions, language, and reading comprehension (6); cerebellum and anterior part of the vermis may suffer hypoplasia and affect motor skills and learning capacity (7). Moreover, proven asymmetry of the hippocampus in FAS children may also affect their memory (8). The degree of structural abnormalities in the brain correlates with the severity of FAS-like facial features, and this in turn, with more serious behavioral problems (9).

According to the World Health Organization, PAE is the main preventable cause of intellectual disability in the western world (10–12). A recent meta-analysis estimated global prevalence of alcohol use during pregnancy to be 9.8% (13). Therefore, PAE-related disorders may lead to major problems for the social environment as well as economic setbacks for the public health system.

Animal models play a key role in the study of FASD by allowing the development of novel diagnostic and therapeutic tools. Researchers have used a great variety of organisms to mimic the physical and behavioral characteristics found in PAE and FASD phenotypes. Inbred strains of rodents are genetically homogenous populations that facilitate result reproducibility and interpretation in studies designed to evaluate the impact of environmental insults such as ethanol. Moreover, the alcohol intake pattern can be more precisely defined (timing and dose), allowing the identification of time-sensitive windows and thresholds of harmful doses during pregnancy. Rodents have been widely used in FASD research to assess the way PAE-related impairments affect metabolic pathways, molecular biology, cell signaling, synaptic plasticity, and cognition during fetal development, promoting the study of variables affected by alcohol exposure at neuroanatomical, neurochemical and behavioral levels (14).

In this review, we focus on rodent FAS-like phenotype neurodevelopmental disabilities, taking into account facial dysmorphology and fetal growth restriction. We examine every stage of brain development, considering changes caused by PAE in different neural cell lineages, molecular pathways and oxidative stress epigenetic variations. We also review the experimental methodologies used to generate rodent FASD-like phenotypes, including advantages and disadvantages of the different routes by which alcohol has been administered. Finally, we revise anatomical and behavioral alterations, as well as the methodologies used to assess these features in murine models.

## FASD-Like Animal Models

FASD studies in humans have common limitations due to the complexity in correctly measuring certain variables such as maternal diet or health, or the volume and timing of ethanol exposure during pregnancy. These difficulties may be resolved by using animal models, simple, effective, and reliable tools for alcohol research. These models are useful for understanding the molecular mechanisms underlying alcohol teratogenicity and for monitoring cognitive and behavioral changes. Animal models also allow assessing different therapeutic approaches in preclinical studies, for initial screening of the compounds and strategies for future human clinical studies.

The invertebrate *Caenorhabditis elegans* is a simple model for development and is commonly chosen to study the effects of ethanol on molecular pathways. However, the embryos develop outside the body, exact ethanol concentrations administered cannot be finely controlled (15), and the way alcohol is metabolized differs substantially from that in humans (15). The zebrafish (*Danio rerio*) has several physiological and genetic similarities with humans (16), which makes it a suitable alternative as model of vertebrate. Regarding the effects of ethanol, there are further advantages: substantial knowledge of all stages of development, short developmental period, and produce large amounts of offspring (17). Zebrafish eggs and embryos are transparent (just like in nematodes) making embryonic development easy to follow, facilitating exposure to alcohol of the embryos during different and precise developmental periods, and easy determination of physical malformations and simple behaviors (16, 18). By contrast, the chorion of the egg acts as a barrier and large volumes of ethanol are necessary to ensure its penetration (17).

Mammals offer significant advantages in the study of brain structures or complex behaviors (19). Although primates could be the gold standard, there are some disadvantages, mainly the long duration of the studies and ethical limitations (19). Rodents are the most employed mammals for FASD research because they are easy to handle, have a short gestational period, and produce large numbers of offspring. Rats offer the advantages of being larger and with a more sophisticated behavior in comparison to mice. Regardless, mice (particularly the C57BL/6 strain) are the most commonly used mammal due to their ease of care, availability of transgenic and disease models, short lifespan, and basic physiology and genetics similar to that of humans. Teratogenic effects of alcohol exposure in mice have been reported, including craniofacial malformations, altered neurogenesis processes, and soft-tissue and skeletal abnormalities (20, 21). The main disadvantage in using rodents for FASD research is that the third trimester equivalent to human development in rodents occurs after birth. Thus, there are differences in the processes of absorption, distribution, metabolism and elimination in rodents in comparison to the human utero, with no influence of the placental barrier. Interestingly, C57BL/6J is the strain with the highest preference for alcohol (22).

In following sections, we discuss details that need to be considered when a murine model is chosen for a FASD study.

### Alcohol Exposure Patterns

Drinking patterns are characterized by the amount and frequency of ethanol taken. This is measured by blood alcohol concentration (BAC) and expressed as weight of alcohol per unit of volume of blood.

Kelly et al. showed that binge-like alcohol exposure is more harmful than non-binge exposure in rat brain development after exposure to the same dose of ethanol. The authors administered doses of 6.6 g/kg/day of ethanol to neonatal rats using artificial rearing, following one of two possible patterns. A continuous pattern (24 h per day) for several days, which resulted in an average BAC peak of 79–97 mg/dL or an acute exposure pattern (8 h per day) for the same period of time, resulting in an average BAC peak of 56–415 mg/dL. Lower brain growth was observed in the acute exposure group in comparison to the continuous pattern (23). Other findings support the hypothesis that lower daily doses of ethanol following a binge-like pattern leads to lower brain weight and cell loss in different brain areas than higher non-binge doses. Three groups of ethanol-exposed rat pups were compared. One group was exposed to 4.5 g/kg/day in a condensed pattern (4 h per day), the second group was exposed to the same dose although administered in a less condensed pattern (8 h per day), and the third group was exposed to a higher dose of alcohol (i.e., 6.6 g/kg/day) administered in a continuous pattern (24 h per day). The resulting average BACs peaks were 361, 190, and 39 mg/dL, respectively. The authors found that pups exposed to 4.5 g/kg/day over 4 h had the lowest brain weight, followed by the second group. The animals that ingested highest doses of ethanol throughout the 24 h had the highest brain weights (24). These results demonstrate that ethanol intake under a binge-like pattern is more harmful than higher doses taken for longer periods of time due to higher BAC peaks in shorter periods of time.

### Control Group

Several studies have assessed the influence of nutritional intake on the teratogenic effects of alcohol (25, 26). Alcohol can replace other nutrients because of its caloric content and may interfere with the absorption of other nutrients due to its inflammatory effects on the stomach (27).

Pair-fed control has been used in some FASD-like animal model experiments since it acts as a calorie-matched control group. A carbohydrate substance (e.g., maltose dextrin or sucrose) is usually employed to replace ethanol-derived calories in the diet (28). A pair-fed group may also allow monitoring a stress condition. On the other hand, the pair-fed group is considered as an imperfect control group, since the pattern of food consumption in this group is different from a physiological intake. Individuals in pair-fed controls consume the assigned food as soon as it is available, creating additional stress associated to food restriction. In addition, in the pair-fed group it is not possible to match the effect of alcohol on the absorption of other nutrients because of its inflammatory effects. Thus, some researchers have suggested the use of a basal control group known as non-handle, *ad libitum*, or *sham*, in which the intake of nutrients resembles the physiological one. This is useful to avoid biases caused by ethanol interference in nutrient absorption (29). Consequently, the use of a pair-fed group and an *ad libitum* control group should be considered as an alternative when designing a FASD murine model study.

### Route of Administration and Dosage Forms

Several modes of ethanol administration methods have been described, particularly in rodent gestation. Ethanol delivery methods directly affect variables such as the alcohol exposure pattern, exact amount of alcohol taken, and generated stress. All these variables must be taken into account during experimental design. Voluntary ethanol feeding and intragastric gavage are the most physiological administration methods. Voluntary ethanol feeding (30, 31) is a safe technique when low stable BAC levels want to be reached. Conversely, intragastric gavage (29) offers a more accurate control of doses and timing, and reaches higher BACs. Inhalation (32) or injection (33) offer some advantages compared to voluntary ethanol drinking and intragastric gavage due to their time efficiency. Artificial rearing is a useful method when the aim of the study is alcohol administration in a third trimester equivalent model (29, 34, 35). Briefly, the choice of method must consider the purpose of the experiment and the researcher's experience. Table 1 [based on a previous review (45)] summarizes the characteristics of the main routes of alcohol administration in rodents and dosage forms, focusing mainly on mice.

**Table 1:** Characteristics of the different routes of ethanol administration in mice.

**Administration route**	**Characteristics**	**Reached BAC**	**Advantages**	**Disadvantages**
Voluntary ethanol feeding	Oral, self-administration. Pre-gestational alcohol consumption is usually introduced (36). Sometimes, ethanol is added to flavored liquid nutritional formulas (Liquid-diet or Sustacal) to allow easy self-administration (37, 38). 10–20% (vol/vol) ethanol solution (36). Possibility of isovolumic and isocaloric pair-fed diet (e.g., maltose-dextrin) in controls (28). Drinking in the dark (DID) procedure mimics binge-like pattern (39).	50–100 mg/dL when ethanol intake is 1–2 g/Kg [10% (vol/vol) ethanol solution]	Prevent the stress caused by other invasive methods. Safe technique. Easy to carry out. Gradual BAC increase. Low, stable BAC levels. Used prenatally.	Lower ethanol BAC achieved compared to other administration routes. Not useful for binge drinking pattern. Difficult control of dose and timing. No proper control of dose in breastfeeding pups. Not recommended postnatally. Lower BAC achieved if saccharin or a sucrose-sweetened solution is added to the alcohol.
Intragastric gavage	Administration of ethanol into the stomach using a gavage needle. Administered volumes <2 mL/100 Kg body weight (40). Allowed alcohol concentration <31.5% (vol/vol) (40). Ethanol dose 2–6 g/Kg/day (28). Ethanol vehicle (water, saline solution, or nutritional formula) (28).	250–300 mg/dL (60 min) for administration doses of 3.8 g/Kg [21% (wt./vol) ethanol solution]	Useful for binge drinking pattern. Accurate control of dose and timing. Reliable high BAC. Useful for pre- and postnatal administration.	Inhibition of suckling behavior in neonates. Stressful procedure for animals. Invasive procedure.
Inhalation	Inhalation chamber filled with ethanol vapor (41). Sometimes, administration of pyrazole to obtain stable BACs (32, 42).	150–250 mg/dl when volatized ethanol (ethanol 95%) is delivered to the chamber at a rate of 10 l/min	Reliable high BAC. Not a stressful technique for animals. Time and labor efficient. Useful for pre- and postnatal administration. Higher BACs in neonates compared to mothers.	Does not mimic the routes of intake in humans. Special equipment required. Interindividual variations.
Intraperitoneal injection	Ethanol solution injection in intraperitoneal space (43). Single or multiple doses for several days during pregnancy.	350–400 mg/dL (60 min) for administration doses of 3.8 g/Kg [21% (wt./vol) ethanol solution]	Rapid increase in BAC. Time efficient. Useful for pre- and postnatal administration. Useful for binge drinking pattern.	Handling-induced stress. Different intake routes in humans. This administration route produces higher BAC in fetuses than other routes using the same PAE. Higher incidence of malformations when used during first trimester equivalent.
Artificial rearing	Intragastric gavage ethanol discharge in pups while being kept in a special setting to mimic maternal environment (29, 34, 44). Placement of gastrostomy catheters.	150 mg/Kg when ethanol solution of 2,5 g/Kg is administered or 420 mg/Kg when ethanol solution of 7,5 g/Kg is administered^*^	Accurate control of dose and timing. Useful for postnatal administration. Mimics human third trimester.	Invasive and expensive technique. Social factors removed due to isolation of pups.

* *Data obtained from experiments with rats (no available data for mice). BAC, blood alcohol concentration; Vol, volume; Wt, weight*.

### Blood Alcohol Concentration

BAC depends on several factors such as dosage, pattern of exposure, metabolic rate, food consumption, tolerance and genetics (46, 47). As mentioned above, BAC peaks are higher when ethanol is administered in a binge-like pattern, even with low doses of alcohol (24). There are several types of methods for measuring BAC: headspace gas chromatography (HS-GC), headspace solid-phase microextraction (HS-SPME), capillary gas chromatography, or enzymatic ADH immunoassays (48). Immunoassays are not as accurate as mass spectrometry and are susceptible to bias by overestimating alcohol concentration due to non-specific interferences. On the other hand, immunoassays are sufficiently accurate, easy to use in any laboratory, and require a small amount of sample (~100 μL). Immunoassays are currently the most commonly used method for determining BACs in peripheral blood.

In animal models, BAC is defined as the amount of ethanol per unit of blood (usually mg/dL), measured when ethanol concentration reaches the highest level in peripheral circulation (49). In rodents, peak concentration is detected between 30 and 150 min (50–100 min in mice and 50–150 min in rats) following administration. The timeline of BAC depends on the administration route, the dosage and the species (rate of ethanol metabolism is 550 and 300 mg/Kg/h in mice and rats, respectively) (50). Severe neurotoxicity is typically linked to binge-like episodes causing higher BACs (i.e., BAC over 300 mg/dl in rats). However, continuous alcohol exposure, reaching lower BAC levels (i.e., BAC below 40 mg/dl in rats) despite higher doses, induces more subtle brain injuries (23, 24).

## Developmental Stages of the Fetal Brain

**Figure 1:**
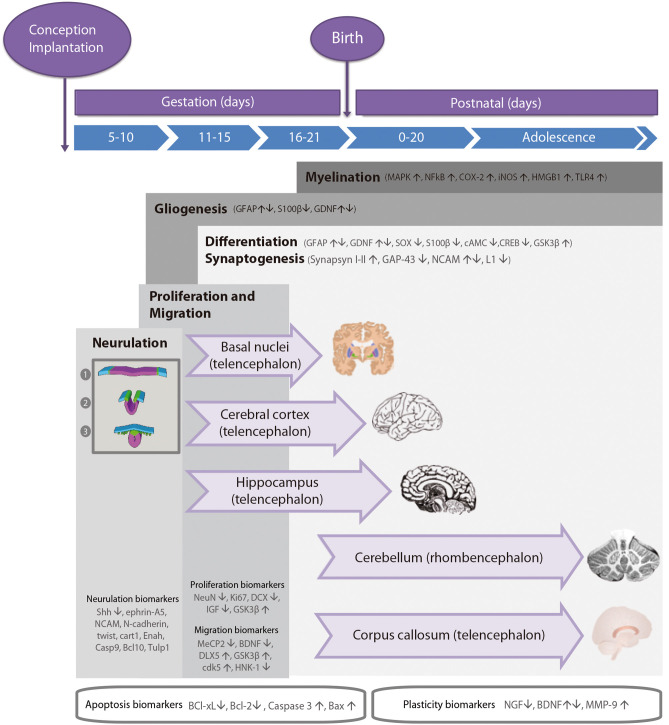
Timeline of neurodevelopmental processes and brain neurogenesis in fetal rodents by areas sensitive to alcohol injury. Proteins in neurulation, proliferation, migration, differentiation, synaptogenesis, gliogenesis, and myelination neurodevelopmental processes. Changes in the levels of these biomarkers (up- or down-regulation) caused by prenatal alcohol exposure are represented by arrows. Stages of neurulation: (1) Neuroectodermal tissues differentiate from the ectoderm and thicken into the neural plate. The neural plate border separates the ectoderm from the neural plate. (2) The neural plate bends dorsally, with the two ends eventually joining at the neural plate borders, forming the neural crest. (3) The closure of the neural tube disconnects the neural crest from the epidermis. Neural crest cells differentiate to form the peripheral nervous system.

**Figure 2:**
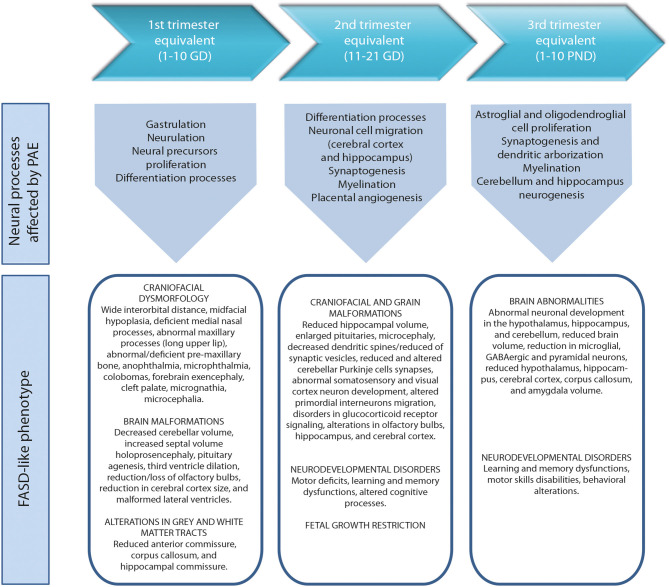
Harmful effects of prenatal alcohol exposure in mice according to human trimesters equivalents (59). First trimester equivalent: cranial dysmorphologies (60, 61), brain malformations (61, 62) and altered gray and white matter tracts (63). Second trimester equivalent: craniofacial and brain malformations (62, 64, 65), neurodevelopmental disorders (66, 67) and fetal growth restriction (68, 69). Third trimester equivalent: brain abnormalities (70–72), neurodevelopmental disorders (71, 73). GD, gestational day. PND, postnatal day.

During the development of the CNS throughout pregnancy, there are vulnerable periods sensitive to environmental insults. PAE affects brain organogenesis differently depending on the dosage, timing, developmental stage (moment), and location of the cell types involved in the biological stages (Figure 1). Key processes such as proliferation (51), migration (52), differentiation (53), synaptogenesis (54, 55), gliogenesis, myelination (56), and apoptosis (57, 58) are altered by PAE leading to congenital abnormalities and functional deficits in the CNS during fetal development (Figure 2) (74, 75).

The anatomies of human and rodent brains show analogous structures and similar stages of development. However, they also exhibit some anatomical and functional differences. Human pregnancy consists of three pre-natal trimesters in which the brain rapidly grows between week 25 and 38. Several differentiation and proliferations processes occur in the third trimester of gestation, with maximum brain growth rate at birth and gradual decrease in early life (59, 76). Rat and mouse pregnancies are shorter than human pregnancies (rats: 21–23 days; mice: 20–22 days) and newborns undergo substantial brain development following birth (56, 57). The first trimester (59) in human pregnancy corresponds to gestational days (GDs) 1–10/11 in rat and mouse. The second trimester equivalent corresponds to GDs 11–21/22 (mice usually give birth on GD 21 and rats on GD 22), and the third trimester equivalent correlates to postnatal days (PNDs) 1–10. The ontogeny of specific behaviors can be used to draw inferences regarding the maturation of specific brain structures or neural circuits in rodents and humans. Despite the similarities between human and rodent brain development it is important to consider that rodents do not exactly mimic the developmental phases of human gestation (Figure 1).

Cellular precursors of the brain and the spinal cord develop through neurulation in early embryogenesis (Figure 1). The cellular fate of neurulation is the formation of the notochord, which defines the primitive axis of the embryo and determines the vertebral system. The neural tube closure starts in the hindbrain area above the origin of the notochord, and continues anteriorly and posteriorly, making a caudal-to-rostral gradient in the developing brain. Neural tube formation finishes at gestational day (GD) 10–11 in rodents (77). Early in the second week of pregnancy (GD 7 in mouse, GD 9.5 in rats), neurogenesis and subsequent cell migration shape specific areas of the CNS in the forebrain, midbrain, and hindbrain, promoting distinct series of developmental processes (77). Therefore, the second critical developmental stage for PAE occurs between GD 5 and 11, implying alterations in organogenesis, neural tube formation and proliferation of neuronal precursors in areas adjacent to the neural tube. High levels of alcohol exposure during this stage not only cause major neural tube defects, but also lead to facial dysmorphologies similar to those observed in children affected with FAS.

The second critical developmental stage occurs between GD 11 and 21. During this period, most CNS areas are involved in distinct differentiation processes and several neuronal cell types emerge and migrate to specific areas of the brain (including the cerebral cortex and the hippocampus; Figure 1). The developmental phase of the different cell lineages varies according to its spatial location in separate brain areas. PAE particularly affects the neurulation, proliferation, and migration processes of the neocortex, cerebellum, hippocampus, and the basal ganglia. The last decisive developmental period occurs from GD 18 to postnatal day (PND) 9 and is characterized by the proliferation of astroglial and oligodendroglial cells, synaptogenesis, and dendritic arborization, which produce an increase in brain weight. At the same time, neurogenesis continues in the cerebellum and the dentate gyrus (DG) of the hippocampus. Alcohol exposure during third trimester induces severe neuronal loss, reactive gliosis, impaired myelination, as well as damage to the prefrontal cortex, hippocampal and cerebellar regions (64, 78, 79).

### Proliferation

Neurogenesis is a highly regulated process whose timing and phases depend on the anterior-posterior gradient in the neuronal axis and the regions of the brain formed during organogenesis. Most cell proliferation processes take place throughout all the stages of neurodevelopment (80), although the most expansive phase occurs in the second half of pregnancy (mice: GD 10–21, rats: GD 11–22). This is a key developmental period due to ethanol toxicity vulnerability of neuronal precursors and brain structures (51), which may cause permanent alterations and profound behavioral deficits. For that reason, the consequences of PAE on proliferation and differentiation processes are assessed not only during fetal development but also later in life. As shown in Figure 1, several biomarkers help identify and evaluate neurogenesis and proliferation processes during neurodevelopment.

NeuN is expressed in nearly all post-mitotic neurons representing a reliable marker of mature neurons (81). This protein may also act as a biomarker of neuronal integrity, as it decreases in brain regions such as the hippocampus following ethanol exposure in rats (81–83). Ki67 has been thoroughly analyzed as a proliferation biomarker during neurogenesis in PAE studies.

Several authors have studied the effect of PAE on different regions of the hippocampus with different results. Some have found a reduced number of granular cells in the DG and pyramidal cells in specific regions of the rat hippocampus after GD 1–GD 20 plus PND 4–10 of ethanol exposure (24, 84), and during the third trimester equivalent (24, 83, 84), with no changes in the number of hippocampal neurons after GD 1–20 ethanol exposure (84, 85). Komada et al. showed a reduced proliferation rate (measured by Ki67) in mouse telencephalon after PAE on GD 6–18 (86). Conversely, West et al. showed an increase in the number of granular cells of the DG in rat hippocampus after ethanol exposure during the third trimester equivalent (87). Thus, early disturbances in proliferation after PAE may differ depending on the developmental period in which exposure to ethanol occurs.

Some of the effects of PAE on the hippocampus can be identified from birth, but others are more subtle and difficult to detect in the early stages. The consequences of PAE on hippocampal cell proliferation and survival in young adult animals are not always persistent (29, 35, 88). Interestingly, no changes in hippocampal cell proliferation (assessed by Ki67 and BrdU), but an increase in immature neurons of adult hippocampus in rats prenatally exposed to alcohol, have been described, probably due to a compensatory mechanism against PAE effects (29). Other authors have shown alterations in cell proliferation [measured by Ki67 (35) and BrdU (35, 88)] and increased neuronal maturation in the DG of the hippocampus in young adult rats prenatally exposed to ethanol. More recently, Gil-Mohapel et al. described significant decreases in adult hippocampal neurogenesis in aged rats after PAE (during first and second trimester equivalent), not previously seen in younger animals. These findings suggest a more conserved neurogenesis capacity in the early stages of life (89). Moreover, Delatour et al. analyzed Ki67 levels in pyramidal cells in adolescent mice exposed to ethanol at GD 13.5–16.5 and showed there were no changes when compared to controls (90). Once again, it seems that alterations in hippocampal neurogenesis vary according to the timing of ethanol exposure.

Coleman et al. examined the long-term effects on adult hippocampal neurogenesis after ethanol exposure in PND 7 in male and female mice. Increased Ki67 levels were found in the DG in males but not in females (70). This reveals gender differences regarding susceptibility to PAE.

PAE also affects the activity of enzymes involved in neurogenesis and proliferation, promoting hippocampal function behavioral disorders. Glycogen synthase kinase-3β (GSK3β) is highly expressed during brain development [from GD18 to PND10 in rats (91) and GD16 to PD18 in mice (92)] modulating different developmental processes such as neurogenesis, differentiation and neuronal survival. GSK3β activation sensitizes neurons to ethanol-induced injury, deregulating cell proliferation mechanisms (93). Increased levels of GSK3β post-PAE activates apoptosis in neural progenitor cells, decreasing neurogenesis and differentiation in immature brains. Additionally, ethanol decreases the insulin-like growth factor (IGF) receptor signaling, affecting neural proliferation and decreasing the transcription of c-myc, c-fos, and c-jun51 in cell cultures (94).

Current evidence indicates that prenatal and neonatal alcohol exposure reduces the number of mature and immature neurons. Interestingly, this reduction is subtle when ethanol exposure is not continuous. Nonetheless, the brain region, developmental stage, and cell type are key factors when analyzing results of the biomarkers in proliferation processes.

### Migration

Migration from the ventricular and germinal layers occurs radially in the medial/dorsal neocortex and tangentially in other regions of the forebrain (95). On GD 5, superficial layers are still not clearly defined (96). On GD 14.5 (mice) or GD17 (rats), the first cell lineages reach the area that will form the laminae of the cortical plate. Throughout the rest of the gestation period until adulthood, the cortical plate gets thicker and more cells migrate from the ventricular zone (97). When proliferation is disrupted, migration is also affected (Figure 1). PAE alters proliferation and migration processes (52), affecting neural crest migration and causing cytoskeletal rearrangements. These phenomena destabilize the formation of focal adhesions in cell lineages, reducing their capacity for directional migration. Moreover, the activity of glycogen synthase kinase 3 (GSK3) and cyclin-dependent kinase 5 (cdk5) modulate microtubule-associated protein 1B (MAP1B) phosphorylation, involved in the regulation of microtubules and actin filaments in neurons, needed in migration processes (98). *In vitro*, ethanol inhibits neurite outgrowth by activating GSK3β (99). Conversely, PAE promotes GABAergic interneuron migration by inducing epigenetic alterations in the methylation pattern of the MeCP2-BDNF/DLX5 pathway. MeCP2 regulates the expression of the brain-derived neurotrophic factor (BDNF), a marker of neuronal plasticity and cellular survival known to influence GABAergic interneuron migration (100). MeCP2 has been shown to regulate DLX5 transcription, a transcription factor involved in the migration and maturation of GABAergic interneurons in mouse models (101). The human natural killer-1 (HNK-1) carbohydrate is also used as a biomarker in migration processes studies involving cranial neural crest cells (102). Results indicate reduced levels of HNK-1 in a model of chick embryos exposed to 2% ethanol, which suggests that PAE may disrupt cranial neural crest cell migration.

Long-term effects of PAE on migration have also been evaluated. Miller et al. describe the harmful effects of alcohol on proliferation and migration in rats prenatally exposed to alcohol. The authors found a delay in migration of early and late-generated neurons in rats following PAE between GD 6 and GD 21. Ethanol blocks neuronal migration, probably by leading to a desynchronization of cortical development that interferes with the establishment of a normal neural network (52). Skorput et al. studied the effects of PAE on GABAergic interneurons in mice. They found an increase in BrdU labeling in the medial ganglionic eminence showing an increase in neurogenesis, as well as an increase in parvalbumin-expressing GABAergic interneurons in the medial pre-frontal cortex in adults. These results support the contribution of GABAergic interneuron migration disorders to persistent alterations in cortical development in adulthood (103).

In summary, migration is a set of complex processes regulated by different molecular pathways that are disrupted in several checkpoints when ethanol exposure occurs.

### Differentiation

Processes of neuroblast differentiation initiate after neuronal precursors have completed their last division and are ready to migrate to a specific area (104–106). Depending on the fate (brain area) of migration, neuronal precursors may differentiate into neurons, astrocytes, or oligodendrocytes (107). The differentiation of the cerebral cortex implies the formation of laminae in the radial domain from the ventricular zone to the pial surface and the subdivision of functional areas in the tangential domain, in rostrocaudal and mediolateral axes. In this process, the laminar fate is determined by cell-to-cell interactions and cell autonomous restriction on their development (104).

Several proteins, used as biomarkers, are involved in the differentiation processes. Doublecortin (DCX) has been studied in depth as an endogenous marker of immature neurons. The effects of pre-natal chronic ethanol consumption on adult neurogenesis (PND 56) has been assessed in C57BL/6J mice, revealing a decrease of DCX in the hippocampus after PAE (82). Quantification of immature neurons labeled with DCX in mouse was lower in the group of individuals exposed to alcohol in the prenatal period compared to controls. Moreover, DCX levels were lower in males than in females (108). Broadwater et al. obtained similar results after PAE by oral gavage on PND28–48, with decreased DCX levels in the DG of adolescent mice. Furthermore, after interrupting ethanol exposure, reduced levels of differentiated neurons in adulthood were found in rats (109). Elibol-Can et al. observed slight changes in the number of granular cells labeled with DCX in hippocampal DG on PND 30. The authors reported a decrease in the volume of the hippocampus in rats after a daily dose of 6 g/Kg ethanol during second trimester equivalent (110). Likewise, Hamilton et al. studied the long term-effects of single or continuous exposure to alcohol during the third trimester equivalent in mice and the effect of voluntary exercise as a therapy. Mice were exposed to ethanol on PND 7 or PND 5, 7, and 9 and DCX measured in adulthood. No differences in DCX levels were found in ethanol exposed groups. Nevertheless, the group exposed to ethanol during PND 5, 7, and 9 showed alterations in the results obtained in Rotarod and passive avoidance behavioral tests, which measure motor coordination and memory, respectively (111). Conversely, Coleman et al. observed increased levels of DCX after ethanol exposure in the DG in adult PND 7 male mice, but not in females (70).

Long-term effects of PAE have also been studied using other biomarkers. Choi et al. assessed the effects of PAE on BrdU levels in adult mice exposed to ethanol during the two trimester equivalents. No differences in neuronal proliferation nor differentiation were found after evaluating BrdU levels (31). Boehme et al. studied BDNF levels of rats exposed to ethanol during the three trimester equivalents. They found no changes in BDNF levels of animals exposed to ethanol in the prenatal period. However, increased BDNF levels were observed in groups assigned to voluntary exercise (35). Gil-Mohapel et al. reported increases in NeuroD levels in adult rats exposed to ethanol during the three trimester equivalents. The increase in differentiation processes are probably due to the increase in immature neurons showed in prenatally exposed groups (29). The changes observed in the differentiation processes in adult rodents exposed to ethanol during the prenatal period vary according to the used biomarker. The increase in neuronal differentiation may occur as a compensation of the cellular loss in fetal life.

Responsive element binding protein (CREB) and cAMP signaling is directly correlated to neurogenesis, differentiation, neuronal connectivity, and plasticity (112). Ethanol exposure disrupts the activity of adenylyl cyclase (AC) reducing cAMP/CREB signaling and therefore altering the differentiation processes during neurodevelopment (112). *In vivo* and *in vitro* studies have shown that acute alcohol exposure enhances agonist-stimulated AC catalytic activity, while chronic alcohol exposure produces adaptive changes in AC (113–115). Additionally, GSK3β over-expression in neural cells disrupts CNS maturation and differentiation processes in mouse at PND 60–120 (116).

The glial cell-derived neurotrophic factor (GDNF) is a growth factor necessary for the development, differentiation, proliferation, and function of midbrain dopaminergic neurons. The GDNF signaling pathway is initiated by the binding of GDNF to its co-receptor, GDNF family receptor-α 1 (GFRα1), which leads to the recruitment of the RET receptor tyrosine kinase. The activation of RET promotes the up-regulation of downstream signaling pathways such as ERK1/2 (117) and P13K (118), firing the activity of dopaminergic neurons. Moderate administration of alcohol increases GDNF expression, exerting a protective function against PAE. However, after acute (binge) ethanol exposure in rats, GDNF expression decreases and its protective function diminished (119). A recent study performed in adult rats exposed to alcohol showed a decrease in DNA methylation as the leading cause of GDNF epigenetic changes following alcohol exposure (120).

Alcohol has deleterious effects on astrocytes despite them being less susceptible than neurons to moderate alcohol consumption (121). Glial cell alterations due to PAE lead to changes in neuron-glia interactions, which causes developmental defects of the brain (122). Glial fibrillary acidic protein (GFAP) is a biomarker of mature astrocytes commonly evaluated in differentiation processes during development. *In vitro* studies using primary cultures of astrocytes from 21-day old fetuses show initial increased values of GFAP levels post-ethanol exposure (123), although these GFAP values decrease after 3 weeks (123). GFAP levels in rat neonates have been shown to increase following ethanol exposure in different brain areas, e.g., the hippocampus, cerebellum, and cortex as per different administration routes (124–126). The results in *in vitro* models suggest different effects of ethanol on astrocytes depending on the neurodevelopmental stage. Moreover, some researchers have found increased GFAP expression associated to gliosis after chronic (moderate) and acute low ethanol exposures, in mice (127, 128). These results indicate a high risk of neurodevelopmental disease in acute PAE or heavy drinkers. Conversely, no changes were observed in GFAP expression after low chronic ethanol exposure (127). S100β is a classical biomarker astrocytes, as the expression levels of S100β in these glial cells is very high. During neurite outgrowth, S100β is also secreted by proliferating astrocytes from cortical neurons. The accumulation of this protein in mature glial cells is associated with microtubule network and neurotrophic activity (129). Reduced levels of S100β were reported in mice after ethanol exposure (130), indicating a depletion in the number of proliferating astrocytes and an impairment in the differentiation processes. Otherwise, Sox2 and Oct4 transcription factors regulate the embryonic stem cell pluripotency and the fate of cell lineages by a narrow range of dose-effect (131). Excess of Oct4 compared to Sox2 leads cells to mesoendoderm differentiation, while the other way round, i.e., higher levels of Sox2, promotes neuroectoderm formation. Ethanol exposure of embryonic stem cells in early differentiation generates imbalances between Oct4 and Sox2, which modifies the cellular fate from neuroectoderm to mesoendoderm, altering the formation of the ectoderm lineage and its derived progenitors. The Oct4/Sox2 imbalance is considered one of the leading causes of developmental delay and anatomical disabilities of the CNS observed in FAS phenotypes (131).

### Synaptogenesis

The developmental process of synaptogenesis involves biochemical and morphological changes in pre- and post-synaptic components. In rodents, maturation of synaptic connections occurs during the postnatal period (Figure 1) (132) and depends on the physicochemical compatibility of pre- and post-synaptic components and the exclusion of inadequate connections. Less harmful effects of alcohol exposure on synaptogenesis have been observed when administered after birth (54, 55), although during neuronal development ethanol seriously alters some mechanisms related to synaptogenesis (54, 55). In a study using a rat model in which individuals were exposed to ethanol 4 weeks before and during pregnancy, the ultrastructural analysis of the cerebellum at PND 7 showed a delayed synaptogenesis and immature appearance of the presynaptic grid (55). PAE affects the expression levels of synaptic proteins such as synapsin 1 and of other proteins of the pre-synaptic (GAP-43, synaptophysin, synaptotagmin) or post-synaptic machinery (MAP 2 and neurogranin). Moreover, ethanol interferes with the function of adhesion molecules such as NCAM (in chick embryo model) (133) and L1 (in mouse model) (134) involved in cell-cell interactions. During the neural processes of migration and morphogenesis, both proteins are involved in the organization and function of synaptic networks, which determine neuronal plasticity. Several studies in animal models (zebrafish) and cell cultures show decreased levels of NCAM after ethanol exposure (135, 136). In other studies, different patterns of NCAM expression were detected according to the developmental stage on which PAE occurs (133) or the NCAM isoform analyzed. For example, the highly sialylated form of NCAM is overexpressed after ethanol exposure but the NCAM 180 and NCAM 140 isoforms appear down-regulated in a rat model (137). Other studies in animal models (mice and rats) have shown down-regulation of L1 following ethanol exposure (134, 138).

### Gliogenesis and Myelination

Glial cells provide nutrients and physical support to neurons and regulate the presence of different proteins and components in the extracellular fluid surrounding neurons and synapses in the brain. They are essential for a normal development and function of the central nervous system (139). Neuroblast migration occurs through a scaffold provided by radial glia (140). Microglia have macrophage functions and astrocytes preserve the ionic and trophic balance of the extracellular medium (141). Oligodendrocytes synthesize myelin, therefore, this cell lineage preserves the myelin sheath and provides trophic support (142). Schwann cells and oligodendrocytes are in charge of the isolation and myelination of neuronal axons (143). Oligodendrocyte progenitor cells proliferate and differentiate into mature oligodendrocytes capable of myelinogenesis (144). Thus, myelination begins later in neurodevelopment than other processes such as proliferation and migration and progresses throughout adolescence in rodents (145, 146). The development of these cell lineages occurs at the same time as neurogenesis in several areas of the central nervous system (141). These lineages are characterized by distinct developmental stages and sequential expression of different developmental biomarkers such as the nerve growth factor (NGF), neurotrophins (NT-3 and NT-4), the brain derived neurotrophic factor (BDNF), and the IGF-1 and IGF-2 factors. The BDNF is one of the most studied neurotrophins. Alcohol alters the levels of BDNF and its receptor tyrosine kinase B (TrkB). PAE induces decreased levels of BDNF in the cortex and in the hippocampus in rats at PND 7–8 (147). Some studies in rats show that TrkB levels decrease in specific brain regions, e.g., in the hippocampus (147, 148) and increase in the cortex (148). The BDNF and its receptor are targets for ethanol damage. Consequently, imbalances between them may contribute to the development of FASD-like phenotypes, even in cases in which the levels of one of them remain unaltered. In general, the up-regulation of these neurotrophic factors show protective effects during development, promoting myelination, cell survival, and neural regeneration in pathological conditions (149).

Lancaster et al. showed that PAE reduces myelinogenesis and its persistence after birth in a rat model (56). Severe impairments in gliosis and a reduction of proteins related to myelin integrity (myelin-associated glycoprotein, myelin basic protein, myelin proteolipid protein, and myelin regulatory factor) was observed in male adult mice exposed to a binge (acute) pattern of PAE during gestation and lactation. This damage was followed by behavioral alterations in executive function and motor coordination (79). These changes could be associated to the behavioral disabilities observed in FASD individuals. It has also been shown that exposure to alcohol activates toll-like receptor 4 signaling pathways (MAPK, NFκB) in a mouse model, leading to an increased expression of pro-inflammatory mediators (COX-2, iNOS, HMGB1) and cytokines. Inflammation processes cause myelinogenesis imbalances, impairments in synaptic links, and activation of the cell death mechanism (150).

### Trophic Support

CNS remodeling is a continuous process that not only takes place during development, but also throughout adulthood in response to environmental influences or genetically programmed events. Alcohol alters synaptic plasticity and neural function (151). Several proteins used as biomarkers participate in neural plasticity processes. Histone deacetylase 2 alters the GluN2A/GluN2B balance [the major subunits of functional N-methyl-D-aspartate (NMDA) receptors] through changes in GluN2B expression, which leads to memory-impairing effects (152). The neurotrophin family of proteins includes NGF, BDNF, NT-3, NT-4/5, and NT-6. It is well-known that NGF and BDNF play important roles in PAE and FASD pathogenesis. Various studies have shown that PAE disrupts neurotrophin pathways, thus affecting the organogenesis and development of brain structures in rodents (153, 154). NGF and BDNF exert their biological effects by activating some members of the tropomyosin-related kinase (Trk) family. NGF activates TrkA and BDNF binds to TrkB (155). Stressful events, neurological injuries, or neuroendocrine alterations in rats increase blood levels of NGF (156). Thus, NGF expression and the functional activity of NGF-target cells in the CNS are seriously affected by alcohol consumption. BDNF regulates neural cell survival and differentiation as well as several functions related to neural plasticity such as learning and memory (157). A recent study found that BDNF levels in the pre-frontal cortex were significantly lower in the group of mice treated with ethanol in comparison to the control group (158). The study concluded that the impairment in learning and memory observed in mice exposed to ethanol was associated to changes in BDNF levels. Stragier et al. showed that chronic and moderate alcohol consumption in C57BL/6J mice promotes a chromatin-remodeling process, leading to up-regulation of BDNF signaling. The authors suggest that this epigenetic regulation is an adaptive process to balance cognitive disorders induced by alcohol (159). Another study in mouse observed a reduction in ethanol dependence after BDNF infusion in the pre-frontal cortex (160), evidencing that BDNF levels in specific brain areas play a role in alcohol dependence. Boehme et al. studied the changes produced by voluntary exercise in hippocampal BDNF levels. Ethanol was delivered by intragastric gavage during the three trimester equivalents and individuals had free access to voluntary exercise on a running wheel during adulthood. Results showed increased BDNF levels in young adult females after voluntary exercise (35). Recent studies suggest that matrix metalloproteinase-9 (MMP-9), a Zn (2)^+^ dependent extracellular endopeptidase, participates in neuronal plasticity, specifically in memory and learning (161, 162). Acute and chronic ethanol exposure up-regulates the MMP-9 levels in the brain, particularly in the medial pre-frontal cortex and hippocampus, in rats (163). The vascular endothelial growth factor (VEGF) is involved in the activity, plasticity and survival of microvessels. Mice prenatally exposed to alcohol have reduced cortical vascular density, affected microvascular structure, and altered expression of VEGF and its receptor. VEGF may prevent microvessel plasticity disorders and death. As a mouse model shows, PAE exerts its deleterious effects on the microvascular network, which suggests that vascular defects contribute to alcohol-induced brain injury (164). *In vitro* studies show that ethanol also alters the expression and function of IGF-I and IGF-II, leading to birth defects such as low head circumference at birth and microcephaly. These insulin-like growth factors are used by the organism as a general signal of cell survival, so that reduced IGF-I or IGF-II signaling by PAE in neurons activates cell death mechanisms by apoptosis or necrosis (165). Other biomarkers such as DYRK1A act as general inhibitors of neural plasticity. Its over-expression in different brain areas due to environmental insults or stress conditions reduces neural plasticity in neurons promoting cognitive problems and intellectual disability (166–168). Recent studies have demonstrated that some DYRK1A inhibitors such as the antioxidant Epigallocatechin gallate (EGCG) improve long-term outcomes related with memory and executive function in individuals with Down syndrome (166–168). Although it is currently under study, the inhibition of DYRK1A could improve the cognitive performance in pathologies associated to the loss of neuronal functions and plasticity, e.g., FASD, Autism, or Down Syndrome (169). Furthermore, EGCG increases NGF expression by downregulation MMP-9. These proteins have been associated with FASD alterations during neurodevelopment (170).

### Synaptic Plasticity

Synaptic plasticity is the process through which long-term changes in synaptic communication occur (171).

Fontaine et al. studied the effect of prenatal exposure to ethanol in a rat model during the two trimester equivalent and PND 21–28 on long-term potentiation, long-term depression, and depotentiation in the medial perforant path input to the DG of the hippocampus. Impairment of long-term potentiation was seen in both males and females, while long-term depression was only observed in males. The results suggest that PAE causes sex specific impairment in synaptic plasticity in long-term depression (172). Wong et al. focused their study in the contribution of microglia in synaptic plasticity. Using a third trimester equivalent mice model, ethanol was injected following a binge-drinking pattern. The authors found a deficit in experience-dependent synaptic plasticity in the visual cortex with no correlation to microglial function (173). Shivakumar et al. administered ethanol to mice at PND 7, and showed that ethanol exposure produces epigenetic changes that inhibit the activation of several synaptic plasticity genes. Coadministration of trichostatin A prevents learning and memory disorders in adult mice (174).

PAE negatively affects synaptic plasticity. Epigenetic changes, as well as damage to the microglia, may partially explain synaptic plasticity disorders in FASD models.

### Apoptosis

Apoptosis is a critical pathway in fetal neurodevelopment. Programmed cell death systematically removes a large number of neural precursors in embryonic structures formed during development. PAE activates and deregulates cell death mechanisms leading to the loss of cell lineages in the hippocampus, basal ganglia, or cerebellum and disappearance of critical structures in the brain such as the *corpus callosum* (57, 58). The activation of apoptosis is produced by an increase of reactive oxygen species (ROS) generated in ethanol metabolism (see section Oxidative Stress). ROS activate intrinsic and extrinsic apoptotic pathways, reducing the expression and function of the anti-apoptotic proteins Bcl-xL and Bcl-2 in a rat model (175). A study using a mouse model shows that the function of the pro-apoptotic effectors Bak and Bax is directly influenced by alcohol due to alterations in mitochondrial membrane fluidity and dysfunctions in mitochondrial respiration, which leads to the activation of the caspase cascade and subsequent generation of the active form of the effector caspase 3 (176). Consequently, some researchers have developed mitochondrial protective strategies to prevent alcohol-induced damage. Certain molecules, e.g., nicotinamide (177), can stabilize mitochondrial membranes while others, e.g., antioxidants, prevent mitochondrial dysfunction induced by the production of ROS following ethanol exposure, in mouse. In addition, ethanol activates specific cell death pathways. More specifically, ethanol induces the phosphorylation of c-jun N-terminal-kinase, a mitogen-activated protein kinase associated with apoptosis and GDNF may interfere with the activation of the c-jun N-terminal-kinase molecular pathway to prevent ethanol-induced apoptosis. Unlike other neurotoxic substances, ethanol does not interfere with the phosphorylation of the extracellular signal-regulated kinases involved in the regulation of cell survival (178).

## Pathophysiology

There are multiple pathological effects derived from alcohol exposure during fetal development depending on the studied organ, region and cell type, as well as the stage of pregnancy in which the fetus is exposed to ethanol (179). The following sections provide a detailed description of the teratogenic effects of PAE.

### Oxidative Stress

**Figure 3:**
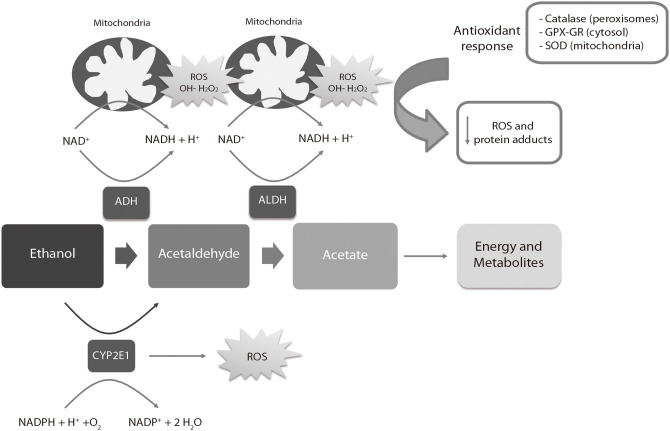
Liver metabolic pathway of alcohol. ADH, alcohol dehydrogenase; ALDH, aldehyde dehydrogenase; ROS, reactive oxygen species; SOD, Superoxide Dismutase; GPX, Glutathione peroxidase; GR, Glutathione reductase. The ADH, ALDH enzymes and the P450 2E1 cytochrome participate in the oxidative metabolism of alcohol. The activity of the P450 2E1 cytochrome in alcohol metabolism and the re-oxidation of NADH via the electron transport chain in the mitochondria results in the formation of ROS. Catalase in peroxisomes, SOD in mitochondria and GPX-GR in cytosol are activated by an increase of oxidative stress to reduce the levels of ROS. High amounts of ROS lead to a down-regulation (negative feedback) of the antioxidant response.

Ethanol is metabolized in the liver of adult individuals via the alcohol dehydrogenase (ADH) and aldehyde dehydrogenase (ALDH) families of enzymes (Figure 3), leading to moderate ROS production, e.g., hydrogen peroxide (H_2_O_2_) and hydroxyl radicals (OH-). ROS are eliminated by endogenous antioxidant mechanisms directed by catalase, superoxide dismutase (SOD) and the antioxidant molecule glutathione (GSH) (180). After a high intake of alcohol, the catalytic activity of ADH and ALDH becomes saturated and an alternative pathway mediated by the cytochrome P450 2E1 enzyme is up-regulated to metabolize ethanol to acetaldehyde, producing high amounts of ROS. ROS-sensing transcription factors, such as the nuclear erythroid 2-related factor 2, activate the oxidative stress response mechanisms when moderate levels of alcohol-derived ROS are present, up-regulating antioxidant enzymes and proteins involved in DNA repair. Imbalances between ROS-producing pathways (following PAE) vs. the endogenous antioxidant and DNA repair mechanisms promotes down-regulation of detoxification pathways (180, 181). The decrease of the antioxidant system affects specific regions of the CNS such as the cerebellum, hippocampus and cortex, as well as the placenta (182, 183). The fetal brain is particularly sensitive to PAE because the ADH isoform expressed in this tissue during development is a class II isoenzyme ADH4. This isoform is less efficient for alcohol catabolism than other isoforms expressed in adults (184). The mechanisms involved in antioxidant response are physiologically downregulated during development (185–188), contributing to brain vulnerability by ethanol. The excess of ethanol also activates the lactate pathway in the fetal liver, generating a deficit of glucose in the bloodstream that affects especially the nervous tissues (189). Imbalances of ROS activate the mechanisms of inflammation (190) mediated by cytokines such as IL-6 or the NLRP3 inflammasome, a multi-protein intracellular complex responsible for processing and secreting the pro-inflammatory cytokines IL-1β and IL-18 (191).

Tissue homeostasis is also affected by ROS (192), causing changes in critical cell functions as signal transduction related to the metabolism of macromolecules (lipids, proteins, RNA, and DNA) (190). AS an example, ROS promote the modification of 8-oxoguanine in DNA during embryogenesis (193, 194), which is corrected by the enzyme oxoguanine glycosylase 1 (195). Calcium homeostasis and protein folding, modification and secretion in endoplasmic reticulum are also altered by ROS, as well as mitochondrial respiration, affecting its morphology and function. Moreover, activation of autophagy, programmed (apoptosis) and non-programmed (necrosis) cell death are also promoted by oxidative stress (192, 196, 197).

Some studies have assessed the long-term consequences of PAE on oxidative stress and the intracellular redox state. Dembele et al. found an relation between continuous administration of PAE with increased levels of oxidative stress in adult rats (PND 90), characterized by high levels of protein carbonyls, lipid peroxides, high expression of SOD, and low levels of GSH (198). Similar results have been reported by other authors, who showed an association between chronic PAE at different concentrations with increased levels of distinct oxidative stress and lipid peroxidation markers in adolescent and adult rodents (199, 200). Chu et al. found a correlation between PAE and apoptotic (p53) and DNA oxidation markers (8-hydroxydeoxyguanosine) in adult rats (200). Brocardo et al. reported depressive and anxiety-like behaviors and high levels of lipid and protein peroxidation in adult rats (PND60) who were given ethanol throughout the three-trimester equivalents (201). Their findings also indicate an association between voluntary exercise, which increased the endogenous antioxidant pathways in brain, and the reduction of oxidative stress and depressive/anxiety-like behaviors. Similarly, binge drinking model of PAE (GD 17–18) increased the levels of lipid peroxidation and oxidative stress, apoptotic activation via caspase-3 activity, and DNA fragmentation, decreasing antioxidant molecules as GSH (202, 203).

### Dysregulation of the Neuroimmune System

Ethanol exposure activates the innate neuroimmune system, causing brain damage and neurodegeneration (150, 204). Alcohol intake triggers the stimulation of microglia and astrocytes, promoting neuroinflammation with the consequent production of pro-inflammatory cytokines and chemokines (e.g., TNF-α, IL-2, IL-6, IL-8, Il-10, IL-1RA, IFN-γ, or MCP-1) (150, 204).

Toll-like receptor 4 and NOD-like receptors have an important function in glial cell stimulation and alcohol-mediated neuroinflammation. Ethanol activates toll-like receptor 4 signaling pathways mediated by NFκB and MAPK, which leads to the up-regulation of cytokines and pro-inflammatory mediators such as HMGB1, COX-2, and iNOS (150). The activation of these inflammation pathways generate severe impairments on synaptic and myelin proteins as well as neural damage (150). Moreover, the increased caspase-3 activity in the prefrontal cortex indicates apoptotic cell death secondary to PAE-related neuroinflammation (205). Regarding myelination and white matter structure, PAE causes neuroimmune changes such as reductions in myelin-associated glycoprotein levels, myelin basic protein and myelin proteolipid protein. Alterations in oligodendrocytes that interfere in the myelination process affecting neural transmission and cognitive development have also been described (79, 205).

### Neurotransmitter Disorders

Neuronal cells and neuroanatomical structures are particularly susceptible to toxic compounds during embryonic development, explained by the high sensitivity of the processes during brain formation. Neuronal damage triggers tissue degeneration by inflammation and massive cell death (apoptosis and necrosis) (182, 206). The loss of some progenitor cell lines seriously affects proliferation, migration, and differentiation of mature neuronal cells, essential to configure the distinct regions of the brain and make them functional (207). The high sensitivity to increases in oxidative stress is the main cause of cell death in these parental lineages. This occurs because they lack the molecules and enzymes required for an antioxidant response, i.e., catalase and superoxide dismutase (181). High levels of ROS affect the mitochondrial function in neurons and leads to the activation of apoptosis (208).

In a prenatal ethanol-exposed brain, differentiation from multipotent glial cells to astrocytes occurs prematurely, preventing the correct completion of migration processes (209). These astrocytes are therefore incorrectly located in the brain, causing motor, and cognitive disorders, promoting cell death of these neuronal groups and triggering harmful effects such as the agenesis of the *corpus callosum* (209). Moreover, primary cultures of hippocampal neurons exposed to ethanol show reduced levels of the glucose transporter GLU1 necessary for the correct growth and development of most cell types present in the brain whose main carbon source is glucose (210). Alcohol also alters the levels of neurotransmitters, namely serotonin, dopamine, and glutamate (211). Exposure to alcohol delays serotonin synthesis, blocking the stimulation of astrocytes and the release of the growth factors needed for proper neurodevelopment (212). Ethanol reduces the number of glutamate receptors (NMDA), which in turn affects other neurotransmitter routes generating important alterations in the transmission of nerve signals (213). The acetaldehyde produced by the metabolism of local ethanol in fetal hippocampus inhibits neurosteroid synthesis and blocks NMDA receptors in pyramidal neurons, contributing to synaptic dysfunction associated with severe alcohol intoxication (214). Furthermore, a recent *in vitro* study with rat brain slices exposed to 70 mM ethanol indicates that the combined overexpression of GABA receptors and inhibition of NMDA receptors results in alcohol-induced neurodegeneration during synaptogenesis (215). Consequently, the administration of single doses of an NMDA antagonist in Sprague Dawley rats causes apoptotic neurodegeneration in young animals, although no impairments were identified in adult individuals. Therefore, the NMDA antagonist acts on the CNS in a similar way ethanol does (216).

PAE has neuroapoptotic effects on the up-regulation of GABAergic transmission and deficit of NMDA receptors. The impairment produced by ethanol on developing neurons depends on the specific neural lineage and is age-dependent. A study performed in mice exposed to moderate amounts of alcohol showed that the subunits of NMDA receptors GluN1 and GluN3A are up-regulated after PAE in the DG. The study also found a decrease of GluN2B levels in the synaptic membrane (217).

### Epigenetic Modifications

During fetal development, epigenetic mechanisms establish the whole pattern of gene expression for the tissues, organs and cell types that constitute the complete organism. These mechanisms involve the methylation of DNA, modifications of N-terminal tails in histones, and the regulation of micro and non-coding RNA.

In DNA methylation, methyl groups (CH_3_), a product of folate metabolism, are added to the cytosines (C) present in the regions known as CpG islands of the DNA helix. This phenomenon is mediated by methyl-transferases (DNMT) and demethylases such as TET2 (218). DNMT3a and 3b set the complete genome expression patterns during fetal development and DNMT1 maintains this pattern in postnatal stages (219). Usually, the clusters of CpG islands match with promoter regions to regulate the expression of the genes involved in a specific pathway or signaling (220). In general, methylation is associated with gene silencing and demethylation with active transcription. Histones regulate the dynamics of chromatin in remodeling processes between heterochromatin, inaccessible to DNA polymerases, and the expanded chromatin (euchromatin) that allows gene expression. Histone structure and function is regulated through different post-translational modifications in their N-terminal tail such as methylation, acetylation, and phosphorylation, mainly in the amino acids lysine, arginine, and serine. All these reversible modifications are carried out by different enzymes such as kinases, acetyl-transferases or methyl-transferases depending on the requirements of the cell (221). Otherwise, non-coding and microRNAs regulate protein translation and mRNA stability, acting post-transcriptionally as inhibitors of mRNA by direct interaction (222).

Ethanol and ROS can modify the activity of methyltransferases and demethylases, directly affecting the global DNA methylation pattern during development. A recent study performed in mice after PAE found 118 differentially methylated regions (DMRs) related to transcription factor binding sites (223). The pathways affected by these DMRs were epigenetic remodeling, hormonal signaling, metabolism, and immune response, revealing persistent occurrence of these alterations in all developmental stages (223). Demethylation following ethanol exposure causes a decrease in IGF-2 levels *in utero*, generating an important delay in growth and leading to skeletal malformations (224).

PAE influences the activity of enzymes such as histone acetyltransferase and histone deacetylase, which modify the composition of the amino-terminal tails in histones (225). Moreover, the acetylation of histones H3 and H4 has been directly related to alterations in the development of the cerebellum, cardiac defects, and hepatic damage (226, 227). Cantacorps et al. (228) showed that a binge PAE pattern in mice alters histone acetylation (lysine 5 and 12 in histone H4) in the pre-frontal cortex and the hippocampus. These long-term epigenetic modifications are associated with cognitive and behavioral impairments in offspring. Ethanol exposure alters the expression pattern during development, affecting non-coding and microRNAs expression (229). PAE generates a decrease in the expression of miR135, miR9, miR21, and miR355. The absence and deregulation of these microRNAs generate early maturation of the progenitor stem cells and an increase in cell apoptosis, producing severe impairments in fetal brain development (230). The high variability of the described epigenetic changes is associated to the dose of ethanol received, the time of exposure and the gestational stage in which alcohol intake occurs.

## Diagnosis of FASD

PAE results in a wide range of phenotypic manifestations and behavioral deficits in the offspring we describe below.

### Craniofacial Anomalies

The key facial features used for a clinical diagnosis of FAS in humans include short palpebral fissures, a thin upper vermilion, and a smooth philtrum (231). Considering previous studies in humans (232, 233), Fang et al. described, for first time in 2009, a validated facial image analysis method based on a multi-angle image classification using micro-video images of mouse embryos. This method, validated later by other researchers, allows discerning between embryos that have been exposed or not to ethanol (232, 233). Rodents provide not only a validated model to study how PAE alters morphogenetic processes, but it also allows making an association between a facial feature alteration and the structure/function in the CNS.

**Figure 4:**
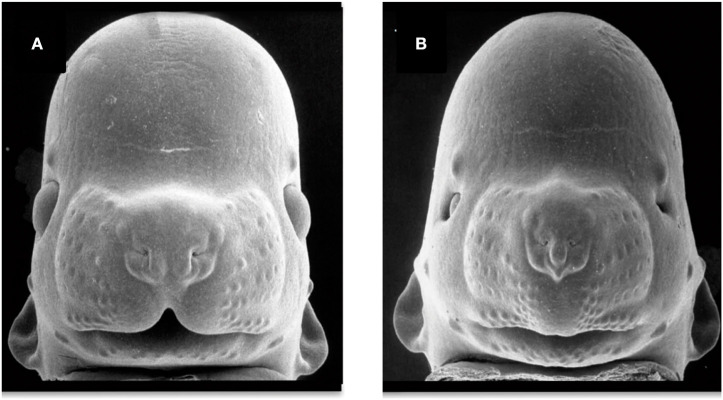
Facial dysmorphology induced by prenatal ethanol exposure. Representative examples of a control animal **(A)** and a fetus severely affected by ethanol exposure **(B)**. FAS-like phenotype **(B)** is defined by microcephaly, short palpebral fissures, thin upper vermillion, and smooth philtrum. FAS-like facial features are induced in the mouse by maternal alcohol exposure on gestational day 7 and 8.5 (equivalent to the third gestational week in humans). Courtesy of Prof. Kathie Sulik, University of North Carolina-Chapel Hill (271) (https://www.teratology.org/primer/fas.asp).

Alcohol exposure during essential periods of embryonic development results in craniofacial dysmorphology (Figure 4). Several studies have used chick and murine PAE models to demonstrate the correlation between craniofacial anomalies, apoptosis induction, and altered migration of neural crest cells (234–236). A series of facial anomalies may present in FASD associated to PAE during the premigratory period of neural crest cells (Figure 2). At this stage, ethanol induces calcium transients that activate CaMKII that mediates the loss of transcriptionally active β-catenin, which produces the apoptosis of populations of neural crest cells. Genetic factors play an important role in the vulnerability to alcohol-induced craniofacial dysmorphology. Sonic Hedgehog signaling, platelet-derived growth factor subunit A, Vang-like protein 2, or ribosomal biogenesis genes are of special importance in neural crest development (237). Studies using FASD-like phenotype rodent models, in which dose and timing of ethanol exposure is controlled, show structural alterations in head and face (238, 239) similar to anomalies observed in humans (240).

Several studies have examined the craniofacial anomalies in FASD-like rodent models. According to Godin et al., intraperitoneal administration of two injections of ethanol at 2.9 g/Kg in mice on GD 7 (equivalent to post-fertilization week 3 in humans), generates a series of facial dysmorphologies similar to those seen in FAS children. These defects include median facial cleft, cleft palate, micrognathia, pituitary agenesis, and third ventricular dilatation and heterotopias (33). However, intraperitoneal alcohol exposure of two 25% dosages of ethanol at 2.9 g/Kg delayed to GD 8.5 in mice produces a different pattern of dysmorphologies such as shortening of the palpebral fissures, mild hypoplasia and shortened upper lip, but a preserved philtrum (60). Variations in FAS-like facial phenotypes depend on exposure timing, implying different facial features when considering this variable (Figure 2). New techniques for FASD diagnosis include methods to identify potentially at-risk individuals based on the identification of subtle and subclinical facial characteristics (241). Scientists have developed a computerized system for detecting facial characteristics using three-dimensional facial imaging and computer-based dense-surface modeling (241, 242). This approach has been compared against standard dysmorphology physical examination for FAS diagnosis revealing high similarities (243). More recently, new techniques based on MicroCT 3D scan performed on pups prenatally exposed to alcohol have been developed (244). This method showed that craniofacial bones might be a reliable and sensitive indicator of PAE in mouse pups exposed to 4.2% alcohol v/v for 2 weeks before the pregnancy and GD 7–16. The same study also confirmed that the neurocranium (cranial skeleton) is more sensitive to alcohol than the viscerocranium (facial skeleton). Other researchers characterized concurrent face-brain phenotypes in mouse fetuses exposed to two 25% intraperitoneal dosages of ethanol at 2.9 g/Kg on GD 7 or GD 8.5 and using MRI imaging and dense surface modeling-based shape analysis (60). Differences in facial phenotype linked to GD of ethanol exposure were found, being more subtle when the exposure was on GD 8.5. Both phenotypes were associated with unique volumetric and shape abnormalities of the septal region, pituitary, and olfactory bulbs. These findings illustrate the need of increasing the current diagnostic criteria to better capture the full range of facial and brain dysmorphology in FASD.

### Brain and Neurobehavioral Deficits

Brain organogenesis is the most severely affected process by alcohol exposure (245) and there is a general consensus in relation to the effects of PAE on the hippocampus, cerebellum, and the *corpus callosum* (246, 247). Important asymmetry of the hippocampus is observed in FAS children, with the left lobe being smaller than the right lobe (8). The cerebellum, associated with balance, coordination and learning capacity, and the anterior part of the vermis develop hypoplasia when exposed to ethanol (7).

The *corpus callosum* is particularly vulnerable to ethanol exposure and, in some cases, may lead to total (agenesis) or partial (hypoplasia) loss of structure (248). The most affected areas of the *corpus callosum* areas are the front (genu) and back (splenium and isthmus), appearing smaller and displaced from the usual spatial location in the brain (6). Basal ganglia is responsible for motor and cognitive abilities, presenting a smaller size in patients with FAS, particularly the area of the caudal nucleus associated to cognitive abilities such as spatial capacity (249). A recent study using three-dimensional surface MRI techniques showed abnormalities in the cortical folding (gyrification) of FASD children. These findings are directly correlated with IQ (250). Future research with MRI techniques to evaluate rodent gyrification may prove to be useful to increase the knowledge on the relationship between cortical development involvement and cognitive disorders in humans.

Broadly, the timing of the ethanol exposure (251) has a clear impact on the CNS and elicits specific brain and behavioral deficits and disorders in motor and cognitive functions (14) (Figure 2).

Different standardized tests in rodents have been used to assess FASD-related abnormalities. As the hippocampus is one of the most damages structures when exposed to ethanol, most studies assessed hippocampal function. Spatial learning is commonly evaluated to demonstrate hippocampal disorders (45, 79, 252). Different authors describe long-term motor coordination impairments, learning and memory deficiencies in adult male mice prenatally exposed to alcohol (79), behavioral effects in rats following short-term PAE (253), or depressive-like behaviors in adult rats exposed to ethanol across the three-trimester equivalents (201). To identify the outcomes of gestational alcohol exposure, a summary of behavioral characteristics after alcohol exposure is needed. Table 2 summarizes the standardized behavioral tests used in rodents to analyze the harmful effects of PAE.

**Table 2:** Standardized experimental methodologies for assessing behavioral effects of prenatal alcohol exposure in murine models.

**Skills affected by PAE**	**Disorders**	**Behavioral test**	**Description**
Motor skills Cerebellum (Purkinje cells) (254)	Motor hyperactivity, poor motor coordination, altered accuracy of saccadic eye movements, and deficits in postural balance are impaired motor skills observed in individuals exposed to ethanol during early life. PAE-related motor deficits are more apparent in early life than in adulthood.	The rotarod (79)	For this test, a bar that can rotate at an accelerated or a fixed speed is used. The latency of the fall of the rodent placed in the bar is measured. Measurements provide an idea of motor coordination.
		Swimming test (255)	It consists of a Perspex tank where rodents are placed and must swim to an escape platform. The sequence is recorded and later analyzed to assess the latency to reach the platform and the number of fore and hind limb strokes.
		Raised beam test	Rodents are placed on the bar and their ability to cross it is measured along with paw slips and traverse time. It provides an indication of their balance.
		Footprint analysis	After their paws are painted or dipped in ink, rodents leave a trail of footprints when they walk or run along a corridor to a goal box. Measurements of stride length, base width, and fore and hind paws overlap give an indication of gait. Automated versions of the task use video processing of footage taken from below the rodents.
Learning and memory Hippocampus (dentate gyrus) (256, 257)	Hippocampal cell loss, altered neuronal morphology, decreased synaptic density, and reduced trophic support.	Simple maze task (258, 259)	A T-maze (or the variant Y-maze) is a simple maze used in animal cognition experiments. It is shaped like the letter T (or Y), providing the subject, typically a rodent, with a straightforward choice. T-mazes are used to study how rodents function with memory and spatial learning by applying different stimuli. The different tasks, such as left-right discrimination and forced alternation, are mainly used with rodents to test reference and working memory.
		Morris water maze (71, 252)	MWM is a test of spatial learning for rodents that relies on distal cues to navigate from a start point around the perimeter of an open swimming box to locate a submerged escape platform. The test allows measuring spatial learning and reference memory.
		Fear conditioning (260)	This test is a form of Pavlovian learning based on the conditioning of an innate response to fear consisting in a complete lack of movements. During an initial phase, the animal is exposed to a conditioned stimulus paired with an aversive experience (unconditioned stimulus). The test measures the fear response in mice replaced in the same location with and without the previous stimulus.
		Object recognition (79, 261)	In an initial session, the rodent is presented with two similar objects. One of the objects is replaced by a new object in the second session. The test measures the amount of time taken to explore the new object.
Executive function frontal cortex and extra-frontal cortex (262)	Disorders in cognitive control of behavior, including basic cognitive processes such as attentional control, cognitive inhibition, inhibitory control, working memory, and cognitive flexibility.	Passive avoidance (263)	Mice learn to inhibit the natural tendency to explore new environments where a negative stimulus was previously obtained. The test consists of a chamber divided in two compartments separated by a gate. Animals are allowed to explore both compartments in the initial phase. In the following phase, they obtain a negative stimulus in one of the compartments. Animals will learn to associate certain properties of the chamber with the negative stimulus. The test measures the latency to cross the gate between the two compartments when the animal is placed in the compartment where no aversive stimulus were obtained.
		Simple maze task (258, 259)	See description in learning and memory.
		Morris water maze (71, 252, 264)	See description in learning and memory.
		Prepulse inhibition (265)	PPI is a neurological phenomenon in which a soft pre-stimulus (pre-pulse) inhibits the reaction of the animal to a subsequent strong stimulus (pulse) often using the startle reflex. Stimuli may be acoustic, tactile, or luminous.
Social behavior (251)	Poor social skills and inappropriate social interactions.	Observation (251)	Feeding difficulties in neonates and lack of parental care. Aggressive behaviors in adults and reversed behaviors between males and females.
Affective behavior (201)	Anxiety- and depressive-like behaviors (201)	Elevated-plus maze (266)	The device is made up of open arms and closed arms, crossed in the middle perpendicularly to each other. Mice have access to all of the arms. The number of entries into the open arms and the time spent in each arm are used as a measure of anxiety-like behavior.
		Forced-swim test (267)	The test is based on the assumption that an animal placed in a container filled with water will try to escape. However, it will eventually exhibit immobility that may be considered a measure of depressive-like behavior.
Olfaction (253)	Injury of the olfactory circuits.	Classical conditioning tasks (268)	An odor is paired with a tempting or aversive stimulus and the response of the animal to the odor is followed by the tester.

*PAE, prenatal alcohol exposure*.

### Fetal Growth Restriction

Ethanol interference with maternal nutrition may differ. As a source of energy, alcohol blocks the absorption of other nutrients, including proteins, and hinders intestinal transport of essential nutrients. Due to its effects on liver, alcohol causes metabolic and nutrient utilization alterations. PAE causes maternal nutritional deficiencies that result in fetal growth deficiencies (26).

PAE also impairs placental angiogenesis (269) and consequently fetal growth restriction (FGR) (270). The growth curves defined by Dilworth et al. are a useful tool to define the frequency distribution of mouse weight. Any fetus with a weight below the fifth centile was considered growth restricted (68). Middaugh et al. characterized the impaired growth of C57BL/6 mice prenatally exposed to alcohol (271) showing the influence of alcohol on fetal growth when administered in the second and third trimester equivalents (271, 272).

Other authors have described the effects of ethanol on trophoblasts and placental permeability. Gundogan showed an altered branching morphogenesis in the labyrinthine zone and the suppression of invasive trophoblastic precursors. This altered process compromised fetal growth and placentation in a dose-response manner (69). The permeability inducer VEGF was up-regulated in mouse placenta after acute alcohol exposure. Permeability was also affected by altered structures in the barriers that separate feto-maternal blood circulation (273). Therefore, altered growth factors in conjunction with malformations of the placental barrier may contribute to placental malfunction and permeability alterations in the feto-maternal barrier.

### Biomarkers for PAE

A biomarker is objectively measured and assessed as an indicator of a normal biological or pathogenic process, or a pharmacologic response to a therapeutic intervention (274). Here we will use the term biomarker as the molecular or genetic indicator that identifies prenatal exposure to ethanol. In murine models, the researcher controls the dose and timing of alcohol exposure and eliminates other variables (e.g., other drugs) that may skew the results. This, and the possibility of getting multiple matrices from the rodents, will allow to obtain appropriate biomarkers for PAE detection.

Some authors have demonstrated a significant decrease of alpha-fetoprotein, a perinatal stress biomarker, in the amniotic fluid of B6J litters exposed to alcohol on Day 8 of gestation, although no differences were found in the B6N substrain (275). Other biomarkers, such as fatty acid ethyl esters (FAEEs), a product of non-oxidative ethanol metabolism and a validated biomarker for PAE, have been detected in mouse heart, liver, placenta, and fetal tissues, 1 h after maternal ethanol exposure. FAEEs were shown to persist for at least seven days in the placenta of the mice and at least 14 days in fetal rat organs (276). Unfortunately, FAEEs cannot be measured in neonatal rodents due to the lack of neonatal hair. By contrast, guinea pigs allow a good approximation since they are born with hair (277). Some authors have shown that FAEE concentrations in exposed offspring samples taken at PND 1 were more than 15-fold higher than their control counterparts (278).

On the other hand, changes in selective neurotransmitters from fetal brains of prenatal alcohol-treated C57BL/6 mice were also observed. Authors showed significant reductions in dopamine, norepinephrine, epinephrine, serotonin, and GABA levels in E13 fetal brains. These results would explain the main causes of abnormalities in brain function and behavior found in fetal alcohol spectrum disorders (279).

In recent years, epigenetic studies in rodents highlight the potential of DNA methylation, histone modification, or non-coding RNA species as biomarkers of PAE. Most of these studies have evaluated general changes for each epigenetic modification. DNA methylation has been the most analyzed marker for PAE-induced epigenetic dysregulation, showing that PAE promotes a global pattern of hypomethylation on fetal DNA during pregnancy affecting critical genes such as *bdnf* (280). Haycock et al. demonstrated that genomic imprinting was also deregulated by PAE, in mouse embryos (281). Low levels of *igf2* expression correlates with PAE due to a specific CpG hypomethylation found in its promoter region (224). *Pomc* expression in neurons, related to stress response, is also reduced by CpG hypermethylation in its promoter (282). The authors suggest that this alteration can be transmitted to offspring, raising the hypothesis that the effect of PAE not only occurs when the fetus is exposed to alcohol but also throughout its whole life and future progeny. Hence, the use of these epigenetic changes using CpG methylations as biomarkers of PAE may be a challenge to consider.

Regarding histone modifications, several studies have found PAE-specific alterations on PAE on H3K9ac, H3K4me2, H3K27me3, and H3K9me2, particularly in the brain. These changes are related to alcohol response mechanisms, e.g., H3K9ac, which has been shown to increase after PAE down-regulates genes related to alcohol response (283, 284). Moreover, the increase of H3K4me2 promotes the up-regulation of genes related to alcohol response (284). A general increase of H3K27me2 was observed in the brain in response to PAE (285), more specifically in the hippocampus and neocortex. H3K9me2 also increased after alcohol exposure suggesting persistent alterations in the expression pattern for a long period, and as such has it being considered as a potential biomarker of PAE (286).

Alterations in non-coding RNA expression following PAE have been assessed in rodents. Results show that PAE causes the suppression of several miRNA such as miR-21 and miR335 in fetal neuronal and progenitor stem cells (230, 287). PAE also suppresses the expression of miR-9 and miR-153 and increases the levels of miR-10a during pregnancy (287, 288). Similar results have been reported in zebrafish after PAE (289) and in a rodent model of alcohol use disorder (290). However, due to the heterogeneity, the low reproducibility and the lack of correlations in the results from the different studies, no consensus has been reached for non-coding RNA, showing the complexity of epigenetic interactions when they are altered by PAE.

Animal models play an important role in the identification and validation of new candidate biomarkers, e.g., selective neurotrasmitters, igf1, igf2, and miRNA. In humans, only the direct biomarkers fatty acid FAEEs, ethyl glucuronide, ethyl sulfate, and phosphatidylethanol in biological matrices are validated to detect PAE (291). The low levels of *igf2* expression after PAE (224) in mouse are in line with the results of a recent publication that compared the levels of IGF I and IGF II in the FASD pediatric population with children objectively non-exposed to ethanol (292). These results highlight the potential use of IGF-I and IGF-II as surrogate biomarkers of the damage induced by PAE. Furthermore, BDNF levels in rodent models are known to be disrupted during acute/chronic and prenatal alcohol consumption (293). Thus, changes of BDNF levels in the meconium, cord blood, or in the mother's/infant's serum are used as potential biomarkers of PAE in humans based on rodents results (293, 294). Moreover, magnetic resonance spectroscopy studies in rodents have shown that neurotransmitter biomarkers of FAS including choline, acetyl choline, N-acetyl aspartate, and glutamate, a precursor for the synthesis of GABA, are significantly reduced in FAS (295). Reduced levels of glutamate, taurine, and N-methyl D-aspartic acid receptor have also been observed in FAS children (296, 297). PAE alters the methionine-homocysteine pathway in rodents and humans. Thus, s-adenosylmethionine, which acts as donor of methyl groups to DNA methylases, may be a promising clinical biomarker of FASD (298).

## Discussion

FASD is a growing problem in our society. Diagnostic difficulties, limited knowledge on the underlying mechanisms of ethanol toxicity, and absence of effective strategies to treat this pathology is a serious medical matter. There is a wide range of FASD-like animal models, and researchers must be very precise and choose the one that suits best the objective of the study. Invertebrates and simple vertebrates allow alcohol exposure and the assessment of physical malformations and simple behaviors at different developmental periods (299). However, when studying brain structures or complex behaviors, mammals offer significant advantages compared to the above mentioned models (19). Further insights on FASD is possible with murine models as they allow evaluating the specificity of dose-dependent alcohol teratogenic effects, the timing and developmental stage of fetuses, brain structures, and complex behaviors. Rodents are useful for exploring promising treatments that may help minimize the effects of PAE. C57BL/6 mice are one of the most commonly used mammals in FASD research because they are easy to handle when searching for malformations or complex behaviors after alcohol exposure. A wide range of precise methodologies and experimental protocols have been performed using these animal models. In this review, we have described and compared these protocols to provide a framework that allows researchers to make the correct choice of animal model in their research project.

Rodents and humans have similar stages of brain development, differing in birth timing. The third trimester equivalent in rodents is postnatal. It seems clear that facial dysmorphology appears when ethanol exposure occurs during first trimester equivalent (33, 60, 236, 300). However, it is difficult to establish the optimal period for ethanol exposure when brain and behavioral alterations are explored, because brain developmental processes occur continuously throughout the second and third trimester equivalent. The criteria to choose an appropriate FAS-like model will depend on the experimental design and research questions, for which different patterns of alcohol exposure, tissue analysis, molecular mechanisms, or cell types assessment will be needed.

The pattern of ethanol exposure and dosages are important parameters. Some studies support the hypothesis that a lower daily dose of alcohol administered in a binge-like pattern results in lower brain weight and greater cell loss than a higher daily dose administered in a non-binge-like pattern, as binge-like patterns lead to higher BAC peaks (24, 301).

It is essential to identify FASD manifestations in the experimental models to reach the objectives of the research. Different biomarkers are involved in the distinct stages of brain development (summarized in Figure 1), which can be of help when studying the effects of ethanol intake during pregnancy. FAS biomarkers are useful to evaluate the effects of experimental therapies. There is a wide spectrum of techniques to assess facial dysmorphology, e.g., the validated facial image analysis method based on a multi-angle image classification using micro-video images of mouse embryos (233), and other experimental novel techniques based on MicroCT 3D (244). To assess neurodevelopment and behavior of FASD-like rodent models, several standardized behavioral measurements have been described (302, 303) (summarized in Table 2), which allow evaluating the different spheres affected by PAE. When choosing the most appropriate test, the skills the researcher wants to analyze must be taken into consideration. The Morris water maze allows an accurate evaluation of a set of cognitive and motor behaviors affected by PAE. It is necessary to underline the existence of other useful alternatives to evaluate these behaviors.

Fetal growth is impaired by the harmful effects alcohol exerts on angiogenesis. Fetal growth restriction may be evaluated through standardized fetal measurements in defined frequency distribution curves (68), or by assessing the placenta using biomarkers and histopathological analysis.

The use of animal models results essential in pre-clinical studies to evaluate the toxicity of potential pharmacological tools. Animal models also provide an insight into the molecular mechanisms altered by alcohol as per the developmental timing of exposure, pattern of exposure, and dosage. Thus, any experimental breakthrough may be directly applied in clinical care to improve the diagnosis and treatment of FASD patients.

This review contains updated information on FAS-like model in rodents, aiming to be a useful reference for researchers working with FASD-like murine models. This is not a systematic review, although we have performed an in-depth narrative review on the topic. We have reviewed methodologies and protocols as per the objectives of the study in order to obtain robust conclusions for future studies. This work will facilitate decision-making when designing a FASD experiment in rodents by exploring and summarizing the currently available information on prenatal alcohol effects in each pregnancy equivalent trimester.

Additional knowledge on cellular, biochemical, genetic and molecular mechanisms, and pathways altered by PAE is necessary. This will allow further experimental research with murine models aiming to improve diagnostic strategies, prevention and treatment for alcohol-related problems.

## Author Contributions

LA and VA-F drafted the initial manuscript and conceptualized the topics, tables, and figures. MG-R, OG-A, VA-F, and LM conceptualized, designed, and coordinated the review. EN-T, LA, RA-L, and MS-D revised the different versions of the manuscript and prepared the figures and tables. All authors critically reviewed the manuscript and approved the final version for publication.

## Conflict of Interest

The authors declare that the research was conducted in the absence of any commercial or financial relationships that could be construed as a potential conflict of interest.
